# Astragaloside IV Alleviates Tacrolimus-Induced Chronic Nephrotoxicity via p62-Keap1-Nrf2 Pathway

**DOI:** 10.3389/fphar.2020.610102

**Published:** 2021-01-18

**Authors:** Ping Gao, Xiaoyi Du, Lili Liu, Hua Xu, Maochang Liu, Xinlei Guan, Chengliang Zhang

**Affiliations:** ^1^ Department of Clinical Pharmacy, Wuhan Children’s Hospital, Tongji Medical College, Huazhong University of Science and Technology, Wuhan, China; ^2^ Department of Pediatrics, Union Hospital, Tongji Medical College, Huazhong University of Science and Technology, Wuhan, China; ^3^ Department of Pediatrics, Maternal and Child Hospital of Hubei Province, Tongji Medical College, Huazhong University of Science and Technology, Wuhan, China; ^4^ Department of Pathology, Wuhan Children’s Hospital, Tongji Medical College, Huazhong University of Science and Technology, Wuhan, China; ^5^ Department of Pharmacy, Wuhan Fourth Hospital, Puai Hospital, Tongji Medical College, Huazhong University of Science and Technology, Wuhan, China; ^6^ Department of Pharmacy, Tongji Hospital, Tongji Medical College, Huazhong University of Science and Technology, Wuhan, China

**Keywords:** astragaloside IV, tacrolimus, chronic nephrotoxicity, p62-Keap1-Nrf2 pathway, oxidative stress

## Abstract

Tacrolimus-induced chronic nephrotoxicity (TIN) hinders its long-term use in patients. However, there are no drugs available in the clinic to relieve it at present. Astragaloside IV (AS-IV) is a saponin extract of the *Astragalus* which is widely used in the treatment of kidney disease. This study aimed to investigate the effect of AS-IV on TIN and its underlying mechanism. Herein, C57BL/6 mice were treated with tacrolimus and/or AS-IV for 4 weeks, and then the renal function, fibrosis, oxidative stress and p62-Keap1-Nrf2 pathway were evaluated to ascertain the contribution of AS-IV and p62-Keap1-Nrf2 pathway to TIN. Our results demonstrated that AS-IV significantly improved renal function and alleviated tubulointerstitial fibrosis compared with the model group. The expression of fibrosis-related proteins, including TGF-β_1_, Collagen I and α-SMA, were also decreased by AS-IV. Furthermore, AS-IV relieved the inhibition of tacrolimus on antioxidant enzymes. The data in HK-2 cells also proved that AS-IV reduced tacrolimus-induced cell death and oxidative stress. Mechanistically, AS-IV markedly promoted the nuclear translocation of Nrf2 and the renal protective effects of AS-IV were abolished by Nrf2 inhibitor. Further researches showed that phosphorylated p62 was significantly increased after AS-IV pretreatment. Moreover, AS-IV failed to increase nuclear translocation of Nrf2 and subsequent anti-oxidative stress in HK-2 cells transfected with p62 siRNA. Collectively, these findings indicate that AS-IV relieve TIN by enhancing p62 phosphorylation, thereby increasing Nrf2 nuclear translocation, and then alleviating ROS accumulation and renal fibrosis.

## Introduction

Tacrolimus is an immunosuppressant drug which is extensively used in organ transplantation and other autoimmune diseases (Hart et al., 2019; Gao et al., 2020). However, tacrolimus therapy is often associated with irreversible nephrotoxicity that eventually progresses to chronic kidney disease (CKD) (Ojo et al., 2003). It is estimated that 16.5% of patients develop tacrolimus-induced chronic nephrotoxicity (TIN) (Chapman, 2011). Despite years of study, there are no drugs available in the clinic to relieve TIN. The most commonly used risk mitigation strategies like monitoring levels to guide dosing and tacrolimus dose limitation were usually associated with increased rejection risk (Sawinski et al., 2016). Hence, it is necessary to discover a drug that can alleviate TIN and is safe for long-term use.

Oxidative stress plays a crucial role in TIN. Tacrolimus can induce renal vasoconstriction, and hence lead to renal hypoperfusion and hypoxia-reoxygenation injury and subsequently to the formation of reactive oxygen species (ROS) or free radicals. Meanwhile, tacrolimus owns a direct effect in the generation of ROS, although the underlying mechanism remains to be elucidated (Naesens et al., 2009). Consequently, the excessive ROS causes irreversible damage of the renal architecture, which is mainly manifested as striped interstitial fibrosis (Lusco et al., 2017). Therefore, anti-oxidative stress therapy may be a potential candidate for TIN (Lim et al., 2017; Luo et al., 2019).

*Astragalus* membranaceus, also known as Huangqi, is a traditional Chinese medicine widely used in the treatment of kidney disease (Zhang et al., 2019). A large number of clinical observations have demonstrated that *Astragalus* is safe for long-term use (Lin et al., 2019). Astragaloside IV (AS-IV), a saponin extract of the *Astragalus* root, is one of the main active ingredients of *Astragalus*. In recent years, some evidence indicated that AS-IV had significant renal protective effect and can attenuate renal fibrosis (Guo et al., 2016; Wang et al., 2020). In addition, AS-IV has been reported to attenuate cisplatin-induced acute kidney injury (Yan et al., 2017; Qu et al., 2020), but its effect on drug-associated chronic nephropathy has rarely been studied.

Anti-oxidative stress is one of the main mechanisms for the renoprotection activity of AS-IV (Zhang et al., 2020). Nuclear factor erythroid-related factor 2 (Nrf2) is the primary defense mechanism against oxidative stress, driving transcription of >300 antioxidant response element-regulated genes (Zhang and Chapman, 2020). Previous researches have suggested that AS-IV could protect renal cells from oxidative stress-induced injury by activating Nrf2 (Chen et al., 2018; Wang et al., 2019a). However, the mechanism by which AS-IV activates Nrf2 remains to be elucidated. Nrf2 is negatively regulated by Kelch-like ECH-associated protein 1 (Keap1) under normal conditions. When the cell is insulted by oxidative stress, Nrf2 dissociates from Keap1, translocates into the nucleus and promotes the transcription of antioxidant genes. Notably, SQSTM1/p62 (referred to as p62 hereafter) has been recently identified as a pivotal regulator of the Keap1-Nrf2 pathway through Keap1 binding (Deng et al., 2020). Whether AS-IV activates Nrf2 through the p62-Keap1-Nrf2 pathway remains to be studied.

Therefore, the objective of our study was to evaluate whether AS-IV can alleviate TIN, and then to investigate the role of p62-Keap1-Nrf2 pathway in the renoprotective activity of AS-IV.

## Methods

### *In Vivo* Experiments

Animal studies were performed in accordance with ethical guidelines for animal studies. All protocols received approval from the Puai Hospital Animal Care and Use Committee (KY2016-006-01). Eight-week old C57BL/6 male mice (SPF Biotechnology Co.,Ltd., Beijing) were housed with a 12-h light/dark cycle with water ad libitum. All mice were placed on a low sodium diet (0.01% sodium diet) for 7 days prior to treatment and continued on this diet throughout the treatment period.

After acclimation for one week, weight-matched mice were randomized into five groups (*n* = 8): control, model, AS-IV low-, middle- and high-dose groups. Mice in control group were subcutaneously given 10 ml/kg/d vehicle (olive oil; Sinopharm Chemical Reagent Co., Ltd., China). Mice in model group were subcutaneously injected with 1.5 mg/kg/d tacrolimus (Aladdin Reagent Co., Ltd., Shanghai, China) for 4 weeks. In addition to tacrolimus, mice in AS-IV groups were simultaneously given AS-IV at 10, 20 or 40 mg/kg/d (KT201901, purity ≥98%, Jintaihe Pharmaceutical Chemical Technology Co. Ltd., Chengdu, China) by oral gavage for 4 weeks. The dosage of AS-IV was determined according to previous researches (Zhou et al., 2017; Cao et al., 2019) and pre-experimental results. At the end of the treatment period, the mice were weighed and then placed in metabolic cages for the measurement of urine volume over 24 h. On the following day, the mice were sacrificed, and blood and the kidney were obtained for further use.

### Kidney Histology

After rinsing with PBS solution, kidney tissue samples were fixed in 10% buffered formaldehyde and embedded in paraffin. Renal histological changes were assessed using hematoxylin-eosin and Masson trichrome staining to analyze renal pathology and fibrosis. Tubulointerstitial fibrosis was defined as a matrix-rich expansion of the interstitium with tubular dilatation, tubular atrophy, tubular cast formation, sloughing of tubular epithelial cells, or thickening of the tubular basement membrane in Masson trichrome-stained tissue sections (Lim et al., 2015). The extent of fibrosis was estimated in minimum of 10 fields per section by counting the percentage of injured area per field using the Image Pro plus software (Media Cybernetics, SilverSpring, United States). Histopathological analysis was performed in randomly selected cortical fields of sections by a pathologist blinded to the identity of the treatment groups.

### Biochemical Assay

The levels of serum creatinine (Scr) and blood urea nitrogen (BUN) were measured with assay kits (Jiancheng bioengineering institute, Nanjing, China). The assay for malondialdehyde (MDA) content was performed according to the protocols of the MDA kit (A003-1-2, Jiancheng bioengineering institute, Nanjing, China). And the enzyme activity of superoxidase dismutase (SOD), catalase (CAT) and glutathione peroxidase (GSH-Px) were examined by the test kits (A001-3-2, A007-2-1, A005-1-2, Jiancheng bioengineering institute, Nanjing, China). The detailed experimental protocols were provided in supplementary materials.

### Western Blotting

The tissue was pelleted by brief centrifugation, resuspended in ice-cold lysis buffer containing 50 mM Tris-HCl, 100 mM NaCl, 1% Nonidet P-40, 10 mM EDTA, 20 mM NaF, 1 mM PMSF, 3 mM Na3VO4 and protease inhibitor mixture, homogenized thoroughly, and centrifuged (12,000 g for 15 min at 4°C). The supernatant fraction was aliquoted and stored at −80 °C before using for Western blot. Western blot analysis procedures were processed according to our previous protocols (Zhang et al., 2018). Images were captured with Micro Chemi (DNR Bio-imaging systems, Israel) and NIH ImageJ software was used to quantify the detected bands. β-actin was used as loading control and all assays were performed at least three times.

### Reverse Transcriptase-Polymerase Chain Reaction (RT-PCR)

Total RNA in kidney tissues of mice was isolated with TransZol reagent (TransGen, Beijing, China). Total RNA (1 μg) in each sample was reversely transcribed into cDNA using a PrimeScript RT Master Mix (Takara Shuzo, Shiga, Japan) according to the manufacturer’s instructions. One μl of the resulting cDNA was used in polymerase chain reaction (PCR). The following primers were employed: Nrf2 primers (forward: 5′-CTC​GCT​GGA​AAA​AGA​AGT​G-3′; reverse: 5′-CCG​TCC​AGG​AGT​TCA​GAG​G-3′), heme oxygenase-1 (HO-1) primers (forward: CAG​GGT​GAC​AGA​AGA​GGC​TAA​GAC, reverse: TTG​TGT​TCC​TCT​GTC​AGC​ATC​AC), NAD(P)H:quinone oxidoreductase 1 (NQO1) primers (forward: 5′-GGA​AGC​TGC​AGA​CCT​GGT​GA-3′; reverse: 5′-CCT​TTC​AGA​ATG​GCT​GGC​A-3′), glutamate-cysteine ligase catalytic subunit (GCLC) primers (forward: 5′-CAC​TGC​CAG​AAC​ACA​GAC​CC-3′; reverse: 5′-ATG​GTC​TGG​CTG​AGA​AGC​C T-3′), Keap1 primers (forward:5′-AAGGACCTTGTGGAAGACCA-3′; reverse: 5′-CCC​TGT​CCA​CTG​GAA​TTG​AT-3′), p62 primers (forward: 5′-ATG​GGA​CGC​TGA​CTC​ACT​GC-3′; reverse: 5′-GAA​GCA​CAG​AAG​AGG​GAG​TCT-3′) and GAPDH primers (forward: 5′-CAA​GGT​CAT​CCA​TGA​CAA​CTT​TG-3′; reverse: 5′- GTC​CAC​CAC​CCT​GTT​GCT​GTA​G-3′). RT-PCR assays were performed on a QuantStudio™ 7 Flex Real-Time PCR system (Applied Biosystems) using SYBR Green MasterMix (ABI). For accurate normalization of quantitative data, quantification was carried out using GAPDH mRNA as an internal standard.

### Cell Culture and Treatment

The human renal tubular epithelial cells (HK-2) were purchased from the American Type Culture Collection (Wuhan Academy of Life Sciences, Wuhan, China). The cells were maintained in DMEM consisting of 10% fetal bovine serum, 100 U/mL penicillin, and 100 U/mL streptomycin. Cells were grown in a humidified atmosphere at 37°C with 5% CO_2_ and 95% O_2_. After 3–5 passages, the cells were trypsin-dissociated and seeded onto collagen-coated 96-well plates at a density of 0.5–1 × 10^5^. The cell viability was measured using Cell Counting Kit-8 assay and normalized as the percentage of control.

### Measurement of Intracellular ROS Level

The total intracellular ROS were determined by the 2′, 7′-dichlorodihydrofluorescein diacetate (H2DCFDA) assay. In brief, HK-2 cells were seeded in a 96-well plate and followed by pretreatment with AS-IV (25, 50 and 100 μM) (Wang et al., 2019a; Wang et al., 2019b) for 30 min, and then co-treatment with tacrolimus (15 μM) at 37°C for 24 h. The cells were loaded with H2DCFDA. The fluorescence images were taken using a fluorescence microscope (System Microscopy IX70; Olympus, Tokyo, Japan) 30 min after. Fluorescent signals intensities of cells were counted using Image-Pro Plus (IPP) software.

### Small Interfering (siRNA) Transfection

For silencing the p62 proteins, HK-2 cells were transfected with 10 pmol of siRNAs for p62 (Invitrogen) using Lipofectamine RNAiMAX (Invitrogen) following the manufacturer’s instructions. And unconjugated control siRNA (Invitrogen) was used for control experiments. The detailed experimental protocols were provided in supplementary materials.

### Statistical Analysis

All data are presented as mean ± SD. The results were statistically evaluated using two tailed Student’s t test, one-way analysis of variance (ANOVA) followed by least significant difference (LSD) post hoc test. Statistical significance was set at *p* < 0.05.

## Results

### Effects of AS-IV on Tacrolimus-Induced Mice Renal Dysfunction

To evaluate the effects of AS-IV on TIN, mice were given AS-IV at the dose of 10, 20 and 40 mg/kg, respectively. Although treatment with tacrolimus for 28 days did not impact animal weight gain or the urine volume, it remarkably increased the levels of SCr and BUN (*p* < 0.01) (Table 1). Whereas, AS-IV at 20 or 40 mg/kg/d restored the levels of SCr and BUN (*p* < 0.01), and no significant difference was found between the two groups. AS-IV at 10 mg/kg/d failed to attenuate tacrolimus-induced renal dysfunction.

**TABLE 1:** Effect of Astragaloside IV on tacrolimus-induced chronic nephrotoxicity in mice (*n* = 8).

	Body weight (g)	Urine volume (ml/d)	Scr (μmol/L)	BUN (mmol/L)
Control	26.5 ± 0.6	0.92 ± 0.09	37.5 ± 5.9	8.5 ± 1.0
Tac	24.5 ± 1.0	1.09 ± 0.17	64.9 ± 11.9**	14.5 ± 3.8**
Tac + AS-IV 10	25.1 ± 0.8	0.98 ± 0.15	55.4 ± 12.2	12.0 ± 3.9
Tac +AS-IV 20	27.1 ± 0.7	1.01 ± 0.10	44.2 ± 6.1^##^	9.0 ± 1.5^##^
Tac + AS-IV 40	26.8 ± 0.6	1.02 ± 0.15	41.1 ± 7.3^##^	8.7 ± 1.8^##^

**Abbreviations:** Scr, serum creatinine; BUN, blood urea nitrogen; Tac, tacrolimus; AS-IV, Astragaloside IV

The values are presented as means ± SD. **p < 0.01 vs. the Control group; ^##^ *p* < 0.01 vs. the Tac group.

### Effects of AS-IV on Tacrolimus-Induced Mice Tubulointerstitial Fibrosis

**FIGURE 1:**
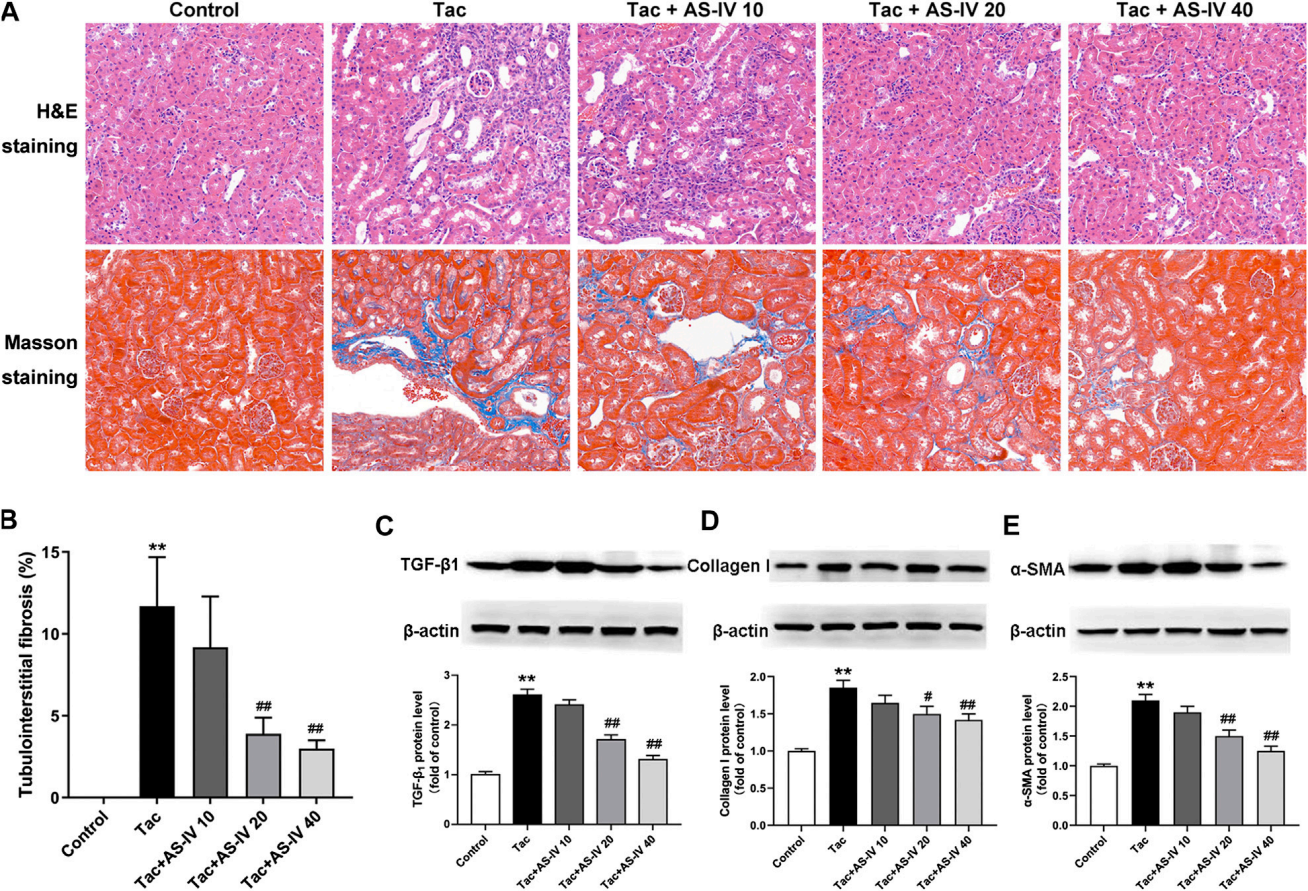
The protective effects of Astragaloside IV from tacrolimus-induced chronic nephrotoxicity *in vivo* (*n* = 8) **(A)** Representative images of HE and Masson staining of renal tissues in mice treated with tacrolimus ± Astragaloside IV for 4 weeks **(B)** Quantitative analysis of the ratio of tubulointerstitial fibrosis in Masson-stained tissues **(C)–(E)** Representative immunoblot image and its quantification of transforming growth factor *β*_1_ (TGF-*β*_1_), Collagen I and α-smooth muscle actin (α-SMA) in the kidney. **p < 0.01 vs. the Control group; ^#^ *p* < 0.05 and ^##^ *p* < 0.01 vs. the Tac group.

HE and Masson trichrome staining of paraffin-embedded kidney tissue indicated that neither inflammation nor tubulointerstitial fibrosis was observed in the control group (Figure 1A). However, treatment with tacrolimus resulted in vacuolar and granular degeneration of tubular epithelial cells, tubular atrophy, tubular cast formation, extensive inflammatory cell infiltration and tubulointerstitial fibrosis. AS-IV at 10 mg/kg/d mildly improved the above-mentioned pathological changes of renal tubules and interstitium, and AS-IV at 20 and 40 mg/kg/d obviously ameliorated it. Further quantitative analysis of Masson trichrome-stained tissues suggested that AS-IV (20 and 40 mg/kg/d) significantly reduced the proportion of tacrolimus-induced tubulointerstitial fibrosis (*p* < 0.01) (Figure 1B). Notably, the renal-protection activity of AS-IV 20 mg/kg/d was not significantly different from that of 40 mg/kg/d (*p* > 0.05). In addition, the biomarkers for TIN were quantified to verify the effects of AS-IV. As shown in Figures 1C–E, tacrolimus significantly increased the expression of TGF-β_1_, collagen I and α-SMA in the kidney compared to the control group (*p* < 0.01). In line with the pathological findings, AS-IV co-treatment (20 and 40 mg/kg/d) remarkably prevented the upregulation of these biomarkers (*p* < 0.05).

### AS-IV Attenuated Tacrolimus-Induced Oxidative Stress in Mice

**FIGURE 2:**
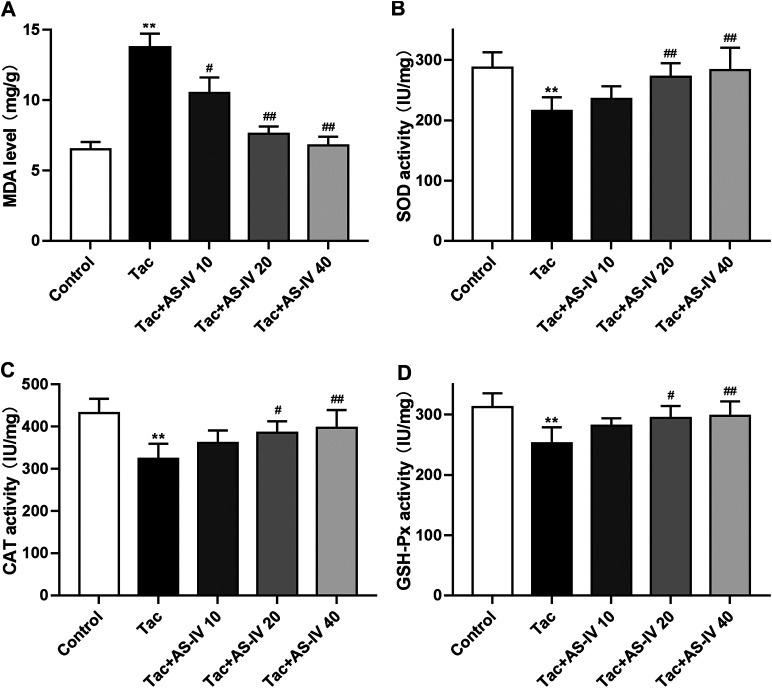
Astragaloside IV attenuates oxidative stress induced by tacrolimus *in vivo* (*n* = 8). Mice were treated with tacrolimus ± Astragaloside IV for 4 weeks, and then renal tissues were taken to evaluate the effects of Astragaloside IV on malondialdehyde (MDA) **(A)**, superoxidase dismutase (SOD) **(B)**, catalase (CAT) **(C)** and glutathione peroxidase (GSH-Px) **(D)** levels. **p < 0.01 vs. the Control group; ^#^ *p* < 0.05 and ^##^ *p* < 0.01 vs. the Tac group.

Tacrolimus caused a significant increased level of MDA as compared to the control group (*p* < 0.01), which was remarkably reduced by the treatment of AS-IV at all three doses (Figure 2A). Besides, tacrolimus administration caused prominent decrease in activity of antioxidant enzymes (SOD, CAT and GSH-Px) in kindey tissue, which was remarkably enhanced by AS-IV treatment at 20 and 40 mg/kg/d (Figures 2B–D). The above *in vivo* data indicated that AS-IV could significantly improve TIN at a dose of 20 mg/kg, so this dose was used for subsequent mechanism studies.

### AS-IV Reduced Tacrolimus-Induced Cell Death and Oxidative Stress in HK-2 Cells

**FIGURE 3:**
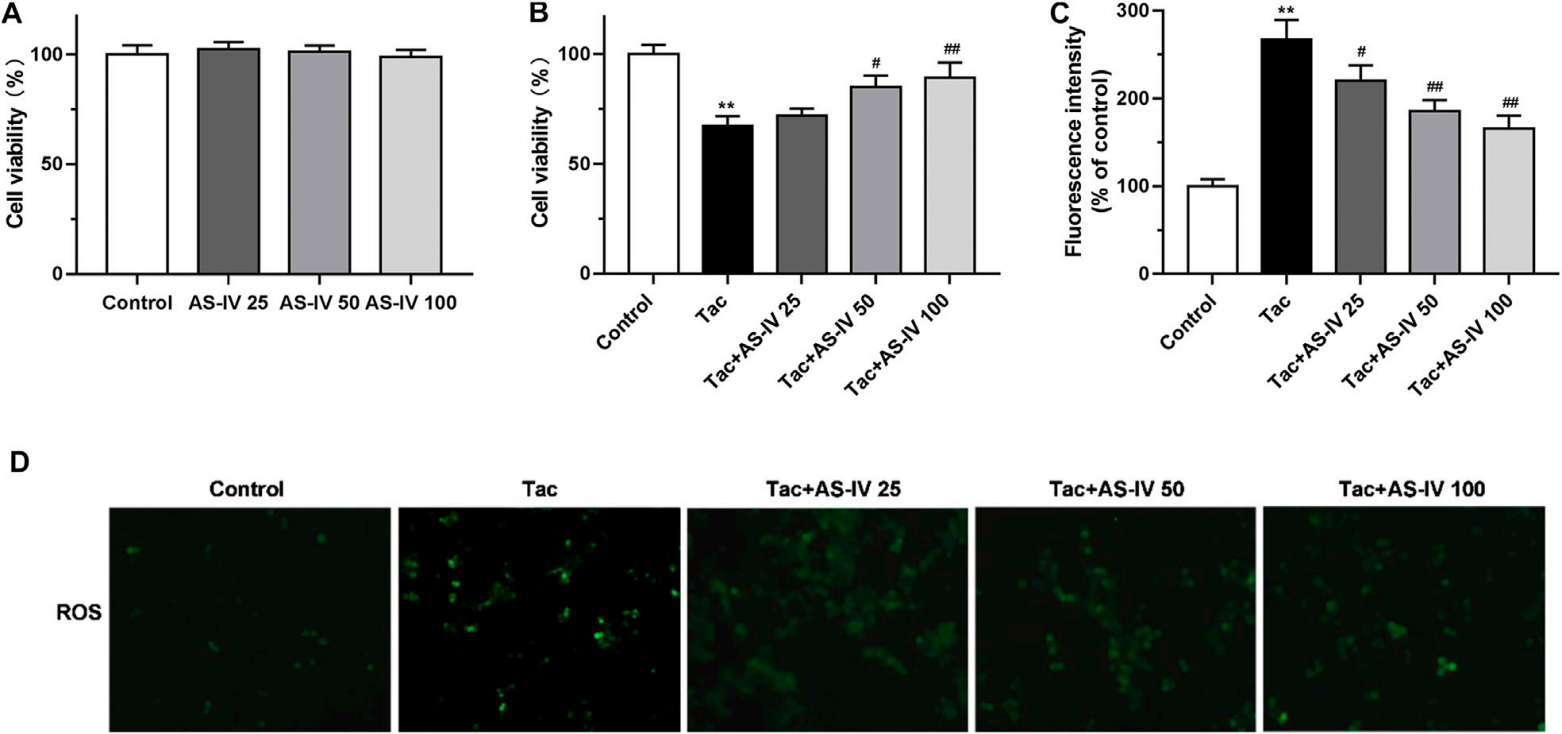
The cell protective and ROS scavenge effect of Astragaloside IV in HK-2 cells **(A–B)** HK-2 cells were treated with Astragaloside IV (25, 50 or 100 μM) ± tacrolimus (15 μM) for 24h, and then the cell viability was measured using Cell Counting Kit-8 assay. The results were calculated from three independent experiments **(C–D)** The levels of intracellular ROS were detected with 2′, 7′-dichlorodihydrofluorescein diacetate (H2DCFDA) assay. Results were calculated by the intensity of eight fields from three independent experiments. **p < 0.01 vs. the Control group; ^#^ *p* < 0.05 and ^##^ *p* < 0.01 vs. the Tac group.

To confirm the protective effect of AS-IV, we evaluated the impact of AS-IV on TIN in HK-2 cells. First, the AS-IV (25, 50 and 100 μM) did not decrease the cell viability (Figure 3A). Compared with the control group, the viability of HK-2 cells treated with 15 μM tacrolimus for 24 h was decreased to 67.3 ± 4.5% (Figure 3B). Whereas, AS-IV at the concentrations of 50 and 100 μM protected cells against tacrolimus-induced injury in a dose-dependent manner. The potential involvement of ROS in the cytoprotective effect of AS-IV against tacrolimus-induced renal injury was subsequently examined. As shown in Figures 3C,D, tacrolimus increased the intracellular ROS levels by 1.67 fold compared with the control group, while AS-IV, especially at 50 and 100 μM, significantly reduced the up-regulation of ROS levels caused by tacrolimus (*p* < 0.01).

### AS-IV Protected TIN via Increasing Nuclear Nrf2 Accumulation *in vivo*

**FIGURE 4:**
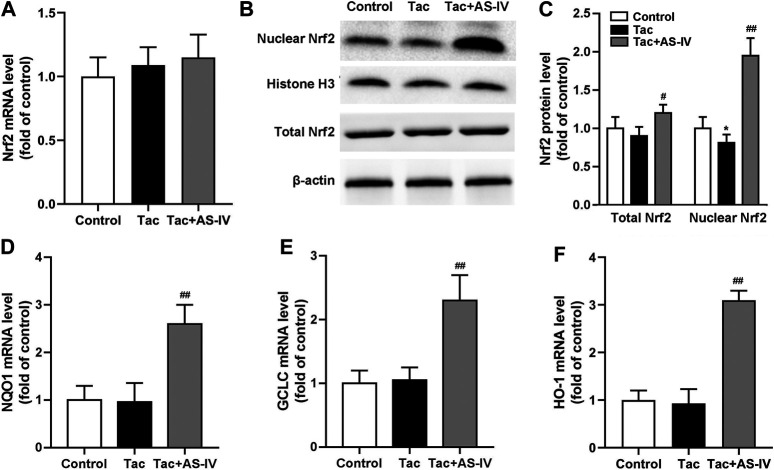
Astragaloside IV induced Nrf2 nuclear translocation and down-stream genes transcription *in vivo*. The mice were treated with tacrolimus ± Astragaloside IV for 4 weeks, and then renal tissues were taken to evaluate the effects of Astragaloside IV on the Nrf2 mRNA levels (*n* = 8) **(A)**, the nuclear and total Nrf2 protein levels (*n* = 6) **(B)–(C)**, the mRNA levels of NQO1 **(D)**, GCLC **(E)** and HO-1 **(F)** (*n* = 8). *p < 0.05 vs. the Control group; ^#^ *p* < 0.05 and ^##^ *p* < 0.01 vs. the Tac group.

Since Nrf2 is crucial in regulating the transcription of plenty of antioxidant genes, we next examined whether the Nrf2 pathway was activated by AS-IV. RT-PCR assays showed that neither tacrolimus nor AS-IV significantly affected the mRNA levels of Nrf2 (*p* > 0.05) (Figure 4A). Tacrolimus hardly affected the protein levels of Nrf2 (*p* > 0.05), while AS-IV significantly raised it (*p* < 0.05) (Figures 4B,C). Moreover, the mRNA expression of HO-1, NQO1 and GCLC, the downstream targets of Nrf2, were significantly upregulated by AS-IV treatment (Figures 4D–F). Considering that Nrf2 activates its target gene expression as a transcriptional factor only in the nucleus, we therefore determine the nuclear translocation of Nrf2 in renal tissue. As shown in Figures 4B,C, the protein levels of Nrf2 in the nucleus was decreased by treatment with tacrolimus (*p* < 0.05), but dramatically elevated by the co-administration of AS-IV (*p* < 0.01). These results indicate that AS-IV abrogates tacrolimus-induced oxidative stress injury may by promoting Nrf2 transfer into the nucleus.

### Nrf2 Was Required for AS-IV to Protect HK-2 Cells From TIN

**FIGURE 5:**
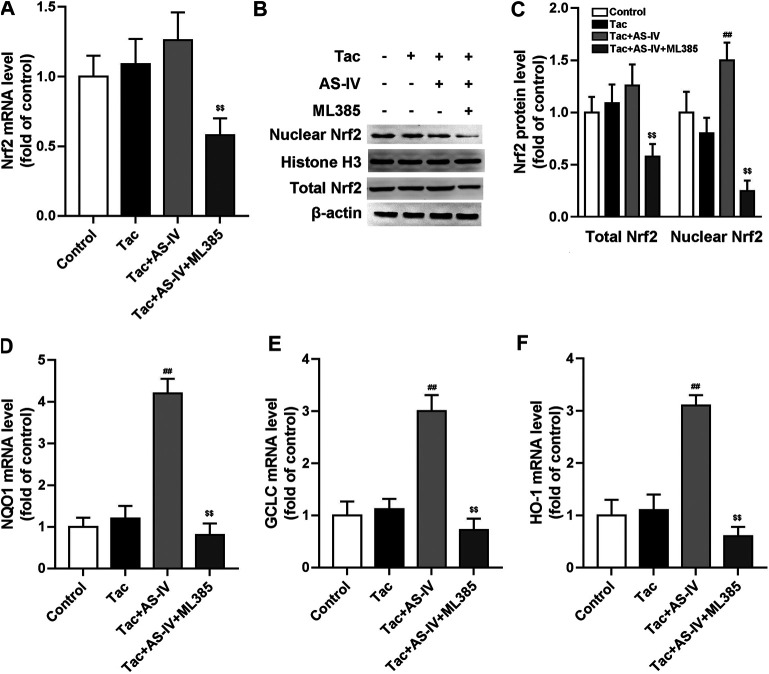
ML385 inhibited Astragaloside IV-induced Nrf2 nuclear translocation and down-stream genes transcription in HK-2 cells. The HK-2 cell were pretreated with AS-IV (50 µM) with or without ML385 (3 µM) for 30 min. Then tacrolimus was added into the cells for 24 h **(A)** The Nrf2 mRNA in HK-2 cells were tested by RT-qPCR assay (*n* = 8) **(B–C)** The nuclear Nrf2 and total Nrf2 protein of HK-2 cell were detected by Western blotting (*n* = 6). The mRNA levels of NQO1 **(D)**, GCLC **(E)** and HO-1 **(F)** were evaluated by RT-qPCR (*n* = 8). ^##^ *p* < 0.01 vs. the Tac group; ^$$^ *p* < 0.01 vs. the Tac + AS-IV group.

To verify whether Nrf2 was dominantly involved in the anti-oxidative stress activity of AS-IV, HK-2 cells were treated with or without Nrf2 inhibitor ML385. Consistent with the *in vivo* data, AS-IV (50 μM) markedly induced nuclear Nrf2 accumulation instead of increasing its mRNA levels in HK-2 cells (Figures 5A–C). The downstream antioxidant genes of Nrf2, such as HO-1, NQO1, and GCLC, were also increased by AS-IV (Figures 5D–F). Whereas, these effects were significantly abrogated by ML385 in the HK-2 cells (Figures 5A–F). Hence, Nrf2 played a critical role in the prevention against TIN by AS-IV.

### AS-IV Increased p62 Phosphorylation and Its Interaction With Keap1, Leading to Nrf2 Activation

**FIGURE 6:**
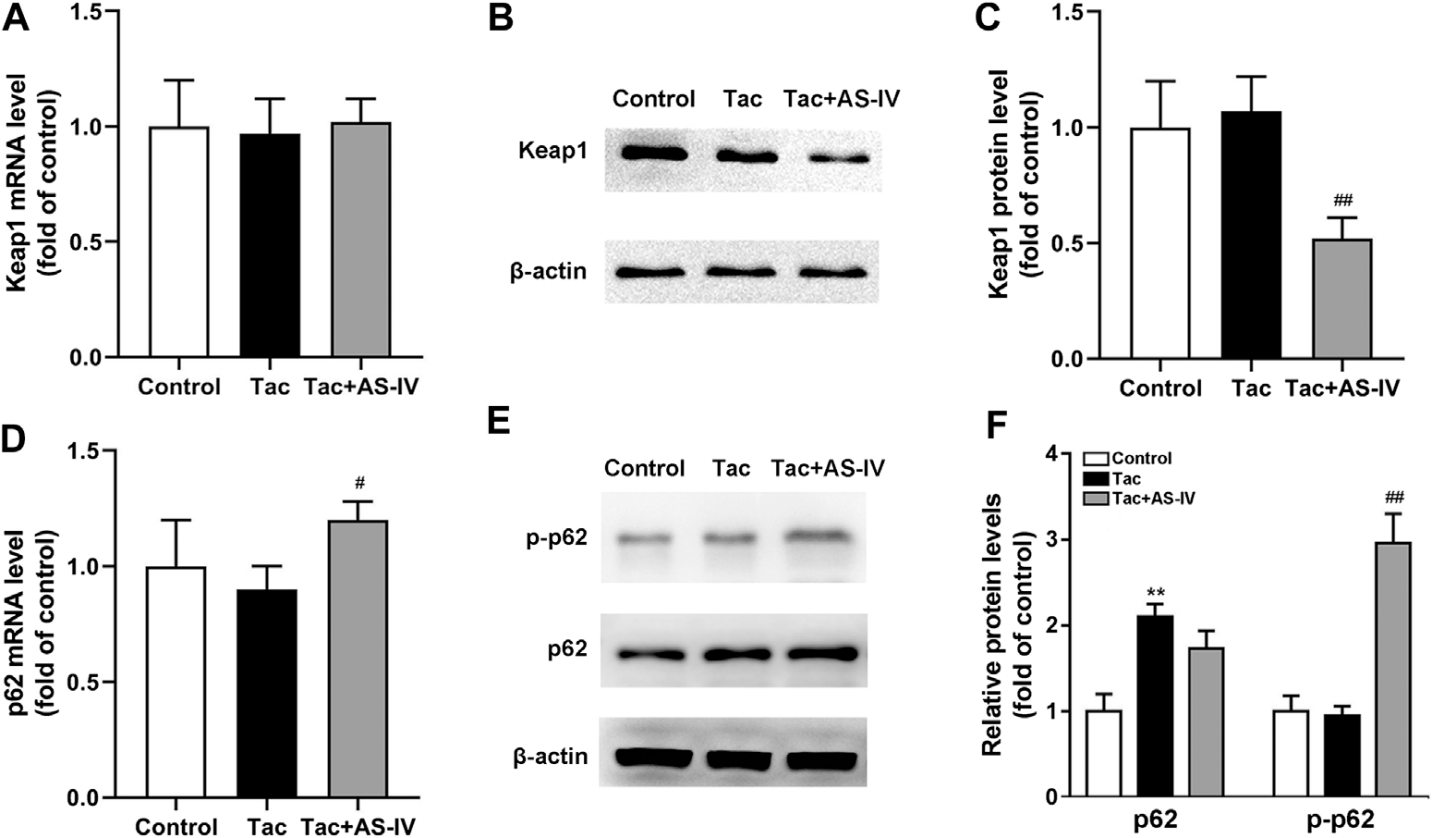
Astragaloside IV increased Keap1 degradation and p62 phosphorylation *in vivo*. Mice were treated with tacrolimus ± Astragaloside IV for 4 weeks, and then renal tissues were taken to evaluate the effects of Astragaloside IV on the Keap1 mRNA levels (*n* = 8) **(A)**, the Keap1 protein levels (*n* = 6) **(B)–(C)**, the p62 mRNA levels (*n* = 8) **(D)** and the p62 as well as phosphorylated p62 protein levels (*n* = 6) **(E–F)**. **p < 0.01 vs. the Control group; ^#^ *p* < 0.05 and ^##^ *p* < 0.01 vs. the Tac group.

AS-IV increased the protein level of Nrf2 without impacting its mRNA, which indicating that AS-IV may function by reducing the degradation of Nrf2. Since Keap1 is the most critical protein that regulates Nrf2 degradation (Cuadrado et al., 2019), the expression of Keap1 was examined. As shown in Figure 6A, mRNA levels of Keap1 were unchanged in both tacrolimus and AS-IV-treated renal tissues. However, the protein levels of Keap1 were obviously decreased after AS-IV treatment compared with that in the model group (*p* < 0.01) (Figures 6B,C).

p62 is well characterized for its ability to recruit and sequester Keap1 to autophagosomes for degradation (Lee et al., 2020). Therefore, the levels of p62 in kidney were subsequently determined. The mRNA levels of p62 were marginally affected by tacrolimus (*p* > 0.05), but significantly induced by AS-IV (*p* < 0.05) (Figure 6D). Unexpectedly, the protein levels of p62 were increased by tacrolimus (*p* < 0.01), but hardly affected by AS-IV (Figures 6E,F). Therefore, we sought an additional mechanism for the decreased Keap1 by AS-IV. Binding affinity between Keap1 and p62 is known to be modulated by phosphorylation of p62 at a specific amino-acid residue, serine 351 (S351) in mice (S349 in humans) (Ichimura et al., 2013; Deng et al., 2020; Lee et al., 2020). Given that, AS-IV may mediate S351 phosphorylation of p62. Thus, we measured the levels of p62 phosphorylation. As shown in Figures 6E,F, tacrolimus didn’t affect the levels of p62 phosphorylation, while AS-IV dramatically increased the phosphorylation of p62 (*p* < 0.01). These results indicated that AS-IV increased the binding between Keap1 and p62 by facilitating p62 phosphorylation.

### Genetic Knockdown of p62 Abrogated the Activity of AS-IV on Nrf2 Nuclear Translocation

**FIGURE 7:**
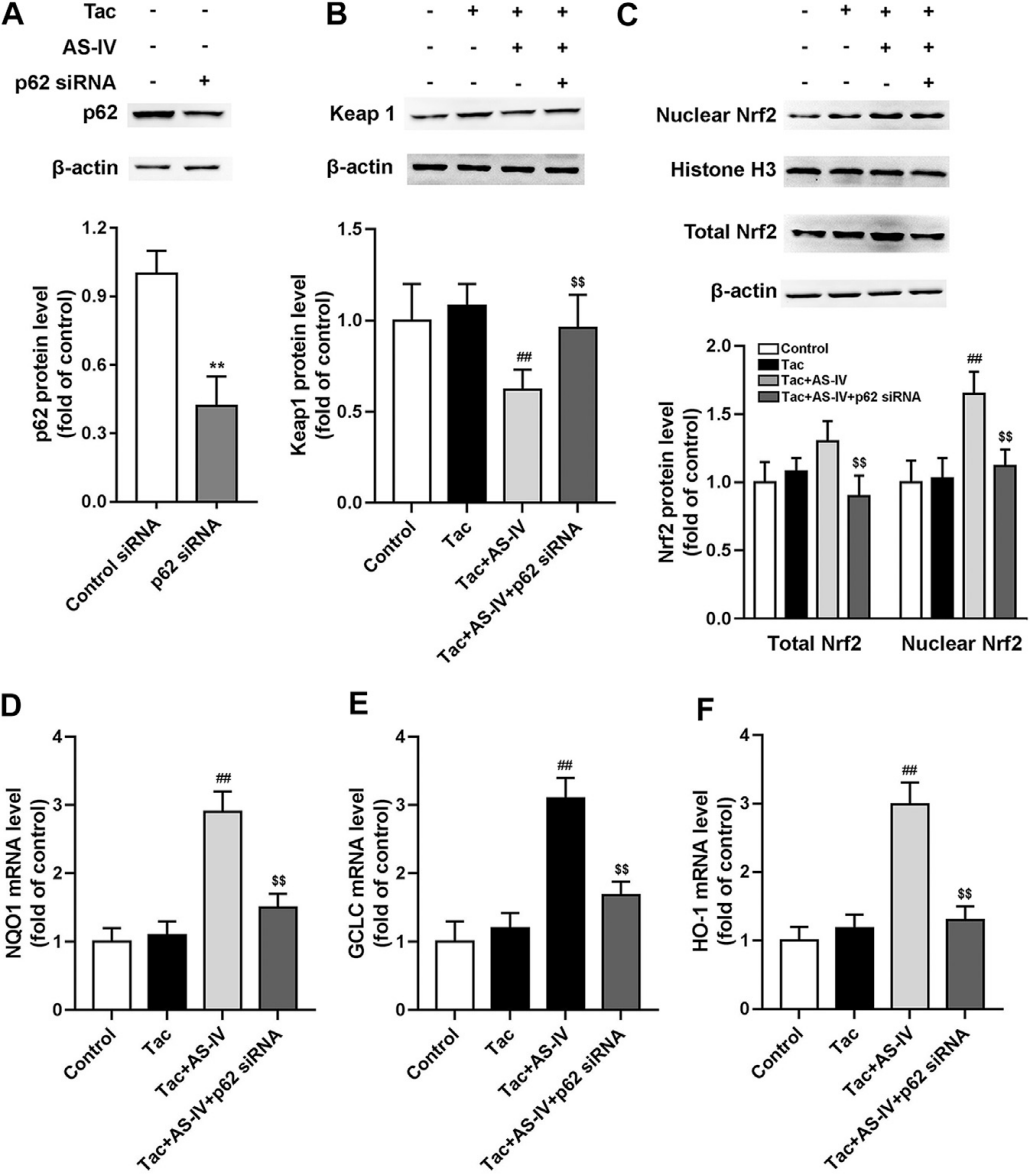
p62 siRNA inhibited Astragaloside IV-induced Nrf2 nuclear translocation and down-stream genes transcription in HK-2 cells via p62-Keap1-Nrf2 pathway. The HK-2 cells were transfected with unconjugated control siRNA or p62 siRNA, and then the protein levels of p62 (*n* = 4) **(A)**, Keap1 (*n* = 6) **(B)**, and the nuclear Nrf2 as well as total Nrf2 (*n* = 6) **(C)** were detected. The mRNA levels of NQO1 **(D)**, GCLC **(E)** and HO-1 **(F)**, were evaluated by RT-qPCR (*n* = 8). **p < 0.01 vs. the Control group; ^##^ *p* < 0.01 vs. the Tac group; ^$$^ *p* < 0.01 vs. the Tac + AS-IV group.

To verify the role of p62, HK-2 cells were transfected with p62 siRNA. After p62 was abrogated, AS-IV failed to decrease the protein level of Keap1, and accordingly Nrf2 nuclear accumulation was cancelled (Figures 7A–C). p62 siRNA also weakened the AS-IV induced up-regulation of antioxidant genes including HO-1, NQO1, and GCLC (Figures 7D–F). Taken together, these gain- and loss-of-function data suggest that p62 is essential for AS-IV-mediated protective effects against oxidative stress-related TIN.

## Discussion

The chronic tacrolimus exposure is associated with an increased risk of CKD, which hinders its long-term use in transplant recipients (Ojo et al., 2003). Despite years of research, no specific treatment for TIN is available. In the present study, we found AS-IV significantly attenuated TIN via minimizing tacrolimus-induced oxidative stress. The anti-oxidative property of AS-IV was causally associated with the activation of p62-Keap1-Nrf2 pathway. These findings suggested that AS-IV was a promising prophylactic or therapeutic option for TIN.

As one of the main active components of *Astragalus*, AS-IV possesses various pharmacological activities, such as neuroprotection, liver and kidney protection (Zhang et al., 2020). Although its oral bioavailability is relatively low (7.4% in beagle dogs and 3.7% in rats) and is mainly eliminated by liver (0.004 1/kg/min), AS-IV owns the highest concentration in liver and kidney (Zhang et al., 2006; Chang et al., 2012). At present, a few studies have explored its role in drug-associated acute nephrotoxicity (Yan et al., 2017; Qu et al., 2020). Nevertheless, the pathogenesis of acute and chronic nephrotoxicity is known to vary considerably (Mehta et al., 2015), so its role in drug-induced chronic nephrotoxicity remains unknown. In this study, AS-IV at 20 and 40 mg/kg/d remarkably reduced the ratio of tubulointerstitial fibrosis (*p* < 0.01) which is a well-recognized marker for TIN (Yu et al., 2019). In addition, tacrolimus-induced increase in SCr (*p* < 0.01), BUN (*p* < 0.01) and fibrosis-related proteins (*p* < 0.05), including TGF-β_1_, Collagen I and α-SMA, were significantly restored by AS-IV. Unfortunately, there has not been a well-recognized effective positive drug used in the researches on TIN until now. Therefore, like most studies (Lee et al., 2018; Lim et al., 2019b; Yu et al., 2019), these markers can only be compared between the treatment group and model group. Despite this limitation, these results can at least indicate that AS-IV was effective for TIN.

AS-IV has been proved to act as a reactive oxygen species (ROS) scavenger to relieve kidney injury in diabetic nephropathy (Du et al., 2018; Wang et al., 2020). Meanwhile, oxidative stress is believed to play a critical role in TIN (Lim et al., 2017; Yu et al., 2019). Hence, we investigated the role of oxidative stress in the renal protection of AS-IV. Our findings indicated that tacrolimus significantly increased the level of MDA (*p* < 0.01) which was a product of phospholipids peroxidation and caused oxidative stress in cells (Lim et al., 2015). And tacrolimus markedly inhibited the activities of antioxidant stress enzymes such as SOD, CAT and GSH-Px (*p* < 0.01). Whereas the MDA level and the antioxidant enzyme activity were obviously restored by AS-IV at 20 and 40 mg/kg/d (*p* < 0.05). The results in HK-2 cells confirmed that tacrolimus significantly increased intercellular ROS levels (*p* < 0.01), while AS-IV significantly reduced it (*p* < 0.05). The *in vivo* and *in vitro* studies suggested that the protective effect of AS-IV against TIN may be associated with antioxidant stress.

It is well documented that the transcriptional activation of antioxidant proteins is dominantly regulated by the redox-sensitive transcription factor Nrf2. Although antioxidant stress has been proved to be effective in ameliorating TIN (Lim et al., 2017; Lim et al., 2019a; Luo et al., 2019), the role of Nrf2 in TIN has rarely been researched. However, we failed to observed that tacrolimus impacted the expression of Nrf2 (*p* > 0.05). Since Nrf2 remains inactive in the cytoplasm under basal conditions and only functions after translocating into the nucleus (Cuadrado et al., 2019), we tested the distribution of Nrf2 in the nucleus. And then we found tacrolimus significantly decreased the protein levels of Nrf2 in the nucleus (*p* < 0.05). AS-IV significantly induced Nrf2 nuclear translocation (*p* < 0.01) and its downstream target genes such as HO-1, NQO1 and GCLC (*p* < 0.01) both in kidney tissues and in HK-2 cells. To further investigate the causal relationship between Nrf2 and AS-IV’s protection against TIN, ML385, an identified chemical compound that specifically binds to the Neh1 domain of Nrf2 and inhibits its downstream target gene expression (Singh et al., 2016), was used. After the pretreatment of ML385, the promotion of AS-IV on Nrf2 nuclear translocation (*p* < 0.01) and its target genes transcription (*p* < 0.01) were abolished. These results confirmed that Nrf2 activation is the key to relieving TIN by AS-IV.

AS-IV increased the protein level of Nrf2 instead of its mRNA level, implying that AS-IV may function by weakening the degradation of Nrf2. It is well-established that Nrf2 is degraded by the Keap1-Cul3 E3 ubiquitin ligase complex through polyubiquitination (Cuadrado et al., 2019). Therefore, we determined the expression of Keap1, and then found that AS-IV remarkably reduced its protein level both *in vivo* and *in vitro* (*p* < 0.01). p62, a critical autophagy-adaptor protein, has a Keap1-interacting region (KIR) domain, which allows p62 to sequester Keap1 into the autophagosomes and promote its degradation via autophagy (Lee et al., 2020). However, the binding affinity of nonphosphorylated p62 for Keap1 was two orders of magnitude weaker than that of Nrf2. Phosphorylation of p62 at Ser351 could enhance p62 affinity for Keap1 binding to a comparable level to that of Nrf2 (Ichimura et al., 2013; Deng et al., 2020). Hence, we detected the levels of p62 and phosphorylated p62, and found that AS-IV markedly raised the latter (*p* < 0.01). Subsequently, siRNA-mediated silencing of p62 was performed to verify the role of p62. In HK-2 cells, p62 siRNA knockdown resulted in a sharply decline of AS-IV-induced Nrf2 nuclear accumulation, which indicated that AS-IV activated Nrf2 in a p62 dependent manner. Interestingly, tacrolimus led to an increase of p62 protein levels, whereas AS-IV marginally decreased its expression. This result cannot be explained by the positive feedback p62-Keap1-Nrf2 loop in which Nrf2 facilitates p62 expression (Lee et al., 2020). Previous research has found tacrolimus could inhibit autophagic flow and resulted in accumulation of p62 (Lim et al., 2019b). Accordingly, we speculate that AS-IV may reduce p62 content by activating autophagic flow, which needs to be confirmed by further research in the future.

In summary, the present study confirms the protective effects of AS-IV against TIN in mice for the first time. This renal protective activity is at least partially attributable to AS-IV-mediated induction of p62 phosphorylation, thereby increasing its competition with Nrf2 for Keap1 binding, and then facilitating Nrf2 nucleus translocation, alleviating ROS accumulation and renal fibrosis. Given the superior therapeutic efficacy of AS-IV in TIN, AS-IV may be developed as a promising candidate drug for the prevention and treatment of TIN.

## Data Availability Statement

The raw data supporting the conclusions of this article will be made available by the authors, without undue reservation.

## Ethics Statement

The animal study was reviewed and approved by Puai Hospital Animal Care and Use Committee.

## Author Contributions

PG, CZ, and XG contributed to the study conception and design. PG, XD, LL, and XG performed the experiment and acquired the primary data. HX, ML, and CZ helped to interpret the data. PG and XD drafted the manuscript. All authors contributed to the article and approved the submitted version.

## Funding

This work was supported by grants from the Wuhan Health and Family Planning Commission (No. WZ18Q03 to PG) and National Natural Scientific Foundation of China (NSFC, No. 81803503 to XG).

## Conflict of Interest

The authors declare that the research was conducted in the absence of any commercial or financial relationships that could be construed as a potential conflict of interest.

## Correction Note

A correction has been made to this article. Details can be found at: 10.3389/fphar.2025.1710116.

## Supplementary material

The Supplementary Material for this article can be found online at: https://www.frontiersin.org/articles/10.3389/fphar.2020.610102/full#supplementary-material


