# Correction: Astragaloside IV alleviates tacrolimus-induced chronic nephrotoxicity via p62-Keap1-Nrf2 pathway

**DOI:** 10.3389/fphar.2025.1710116

**Published:** 2025-10-27

**Authors:** Ping Gao, Xiaoyi Du, Lili Liu, Hua Xu, Maochang Liu, Xinlei Guan, Chengliang Zhang

**Affiliations:** ^1^ Department of Clinical Pharmacy, Wuhan Children’s Hospital, Tongji Medical College, Huazhong University of Science and Technology, Wuhan, China; ^2^ Department of Pediatrics, Union Hospital, Tongji Medical College, Huazhong University of Science and Technology, Wuhan, China; ^3^ Department of Pediatrics, Maternal and Child Hospital of Hubei Province, Tongji Medical College, Huazhong University of Science and Technology, Wuhan, China; ^4^ Department of Pathology, Wuhan Children’s Hospital, Tongji Medical College, Huazhong University of Science and Technology, Wuhan, China; ^5^ Department of Pharmacy, Wuhan Fourth Hospital, Puai Hospital, Tongji Medical College, Huazhong University of Science and Technology, Wuhan, China; ^6^ Department of Pharmacy, Tongji Hospital, Tongji Medical College, Huazhong University of Science and Technology, Wuhan, China

**Keywords:** astragaloside IV, tacrolimus, chronic nephrotoxicity, p62-Keap1-Nrf2 pathway, oxidative stress

In the published article there were mistakes in Figures 3D, 6E as published. In Figure 3D, the image of Tac group (the second group) was misused. In Figure 6E, the Western blot of p62 was misused. The corrected Figures 3, 6 appear below.

**FIGURE 3:**
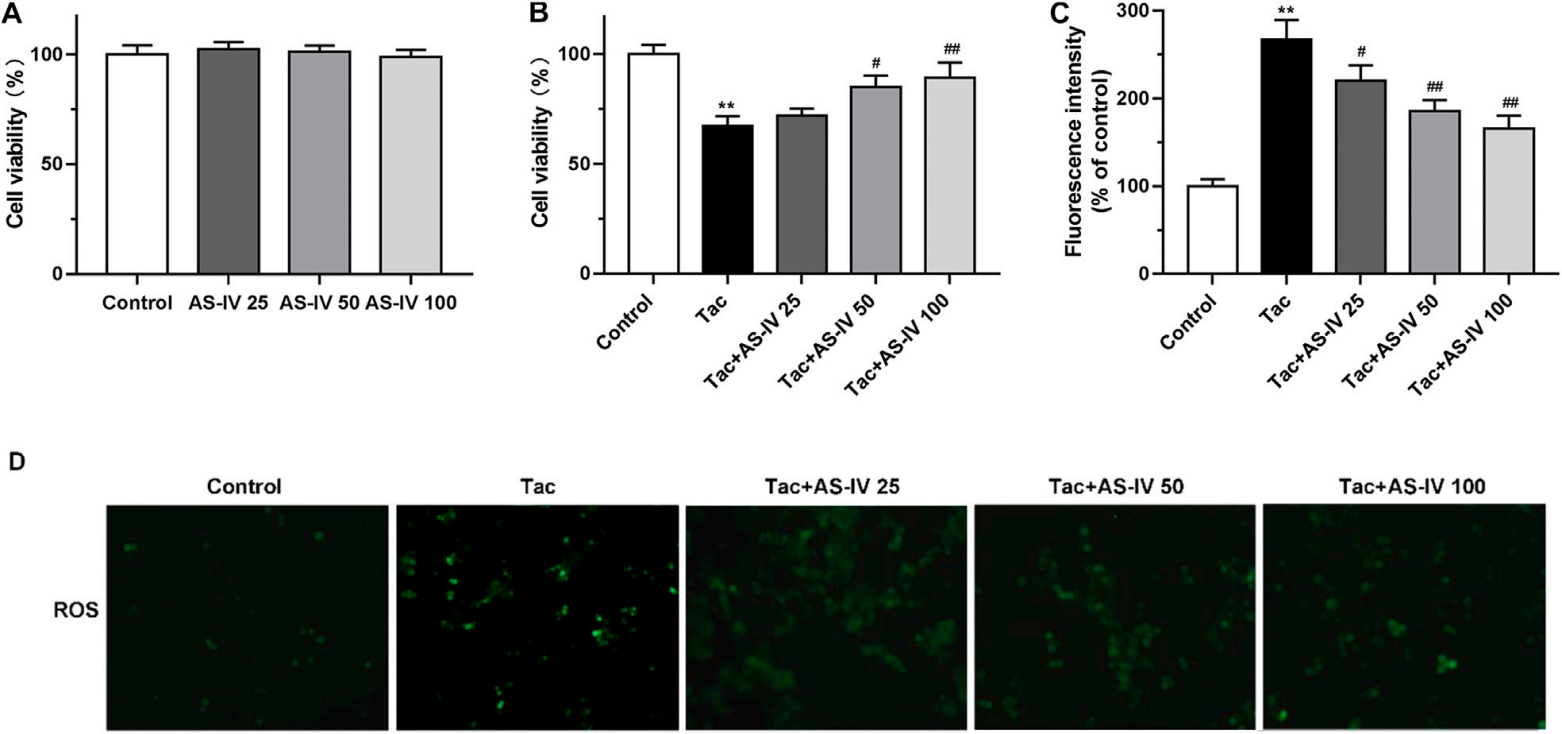
The cell protective and ROS scavenge effect of Astragaloside IV in HK-2 cells **(A–B)** HK-2 cells were treated with Astragaloside IV (25, 50 or 100 μM) ± tacrolimus (15 μM) for 24h, and then the cell viability was measured using Cell Counting Kit-8 assay. The results were calculated from three independent experiments **(C–D)** The levels of intracellular ROS were detected with 2′, 7′-dichlorodihydrofluorescein diacetate (H2DCFDA) assay. Results were calculated by the intensity of eight fields from three independent experiments. **p < 0.01 vs. the Control group; ^#^ *p* < 0.05 and ^##^ *p* < 0.01 vs. the Tac group.

**FIGURE 6:**
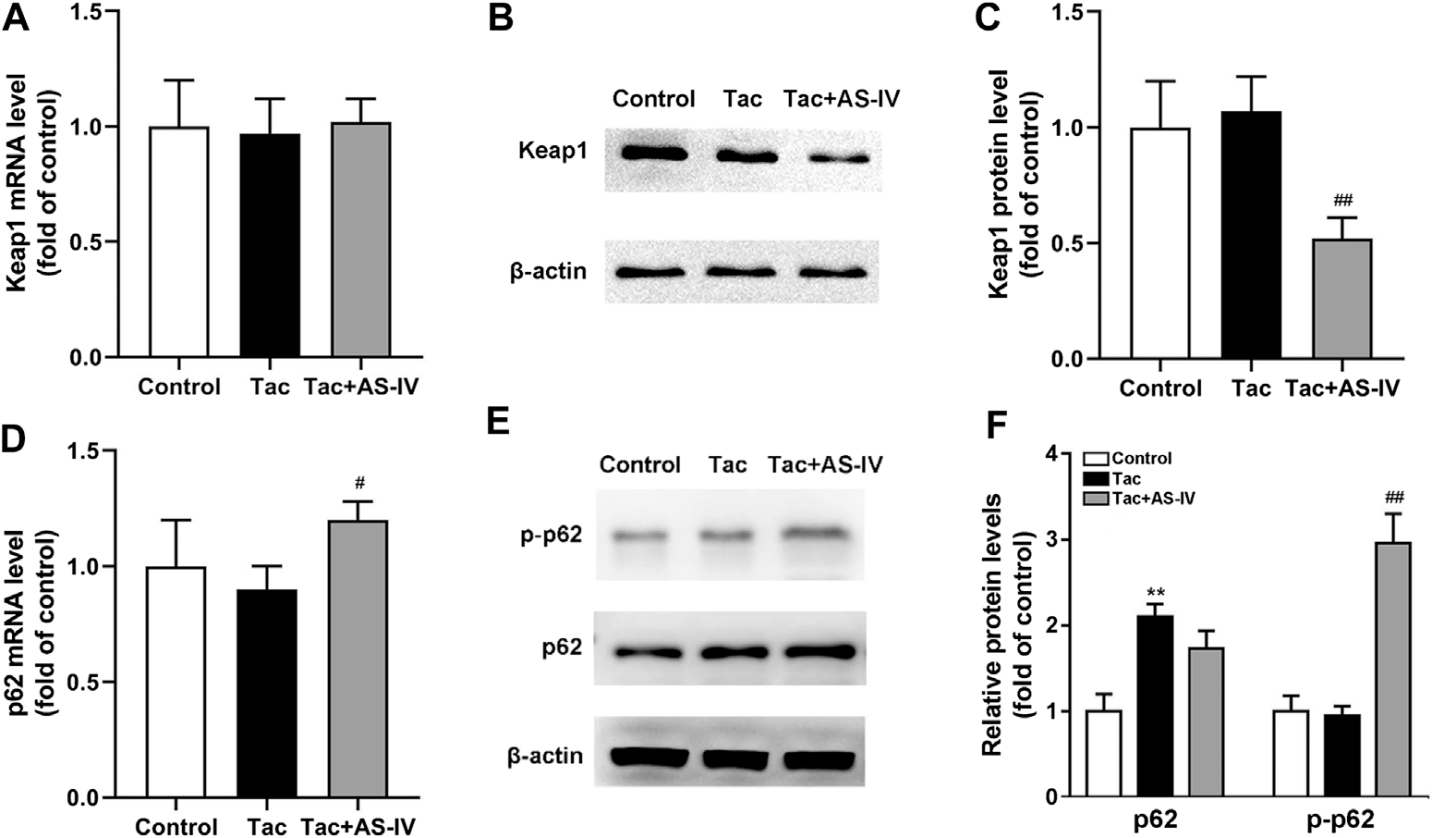
Astragaloside IV increased Keap1 degradation and p62 phosphorylation *in vivo*. Mice were treated with tacrolimus ± Astragaloside IV for 4 weeks, and then renal tissues were taken to evaluate the effects of Astragaloside IV on the Keap1 mRNA levels (*n* = 8) **(A)**, the Keap1 protein levels (*n* = 6) **(B)–(C)**, the p62 mRNA levels (*n* = 8) **(D)** and the p62 as well as phosphorylated p62 protein levels (*n* = 6) **(E–F)**. **p < 0.01 vs. the Control group; ^#^ *p* < 0.05 and ^##^ *p* < 0.01 vs. the Tac group.

The original article has been updated.

