# Supplementary material for: Astragaloside IV Alleviates Tacrolimus-Induced Chronic Nephrotoxicity via p62-Keap1-Nrf2 Pathway
Source: Front Pharmacol. 2021 Jan 18;11:610102. doi: 10.3389/fphar.2020.610102 (PMC7848072; doi:10.3389/fphar.2020.610102)
Supplement: Supplementary file 1 [file Table1.DOCX]

TABLE S1. Effect of Astragaloside IV on tacrolimus-induced oxidant stress *in vivo* (n=8).

|  | MDA  (mg/g) | | SOD  (IU/mg) | | CAT  (IU/mg) | | GSH-Px  (IU/mg) | |  |
| --- | --- | --- | --- | --- | --- | --- | --- | --- | --- |
| Control | | 6.5±0.5 | | 287.6±25.1 | | 432.3±33.8 | | 312.5±22.8 | |
| Tac | | 13.7±1.0** | | 215.3±22.9** | | 323.4±35.8** | | 252.1.5±26.8** | |
| Tac + AS-IV 10mg/kg | | 10.5±1.1^#^ | | 235.1±21.5 | | 361.1±30.1 | | 281.2±12.5 | |
| Tac + AS-IV 20mg/kg | | 7.6±0.5^##^ | | 272.2±22.3^##^ | | 385.5±27.2^#^ | | 294.1±20.1^#^ | |
| Tac + AS-IV 40mg/kg | | 6.8±0.6^##^ | | 283.4±37.1^##^ | | 397.1±42.1^##^ | | 297.6±24.2^##^ | |

Abbreviations: MDA, malondialdehyde; SOD, superoxide dismutase; CAT, catalase; GSH-Px, glutathione peroxidase; Tac, tacrolimus; AS-IV, Astragaloside IV

The values are presented as means ± SD. ^**^*P* < 0.01 versus the Control group; ^#^*P* < 0.05 and ^##^*P* < 0.01 versus the Tac group

**Supplemental Methods**

**Biochemical Assay**

Kidney tissues were homogenized in phosphate buffer (50 mM, pH 7.4) and sonicated at 4 °C. The tissue homogenate was prepared for testing the activities of enzymes, cytosolic fractions which were obtained after centrifugation at 10,000 × g.

Malondialdehyde (MDA) content was detected by the method of Ohkawa (Ohkawa, Ohishi and Yagi 1979), the colorimetric reaction of thiobabituric acid (TBA). Total lipids were extracted with chloroformmethanol (2:1, v/v). The mixture was finally made up to 4.0 ml with distilled water, and heated at 95°C for 60 min. After cooling with tap water, 1.0 ml of distilled water and 5.0 ml of the mixture of n-butanol and pyridine (15: 1, v/v) were added, and the mixture was shaken vigorously. After centrifugation at 4000 r-pm for 10 min, the absorbance of the organic layer (upper layer) was measured at 532 nm.

Superoxide dismutase (SOD) activity was measured by monitoring the inhibition of ferricytochrome-c reduction using xanthine-xanthine oxidase as the source of O_2_^·-^ according the method of McCord and Fridovich (McCord and Fridovich 1969). One unit of SOD is calculated as the amount of protein required to inhibit 50% of the SOD independent cytochrome ‘c’ reduction.

Catalase activity was assayed by the method of Aebi (Aebi 1984) with an commercial test kit. The activity was expressed as μmol H_2_O_2_ consumed/min/mg protein (e = 43.6 mM^−1^ cm^−1^). Briefly, 1 ml of the reaction mixture containing a 50 μl sample was mixed 900 μl phosphate buffer (0.1 M, pH 7.0) and 50 μL of H_2_O_2_ (8.8 mM) and the decrease in absorbance (at 240 nm) was followed for 5 min at room temperature using a UV–vis spectrophotometer.

The activity of glutathione peroxidase was determined using t-butyl hydroperoxide as the substrate according to the method of Flohe and Gunzler(Flohé and Günzler 1984) and the activity was expressed as micromoles of NADPH oxidized/min/mg protein (e340 = 6.22 mM^−1^ cm^−1^). Briefly, the reaction mixture containing 50 μl sample, phosphate buffer (0.1 M containing 0.5 mM EDTA), 100 μl Glutathione reductase (0.24 U), 100 μl GSH (1 mM), 100 μl NADPH (0.15 mM) was incubated at 37 °C for 3 min and the reaction was initiated by the addition of 100 μl tbHP (0.12 mM). Change in absorbance at 340 nm was followed for 5 min spectrophotometer.

**Cytoplasmic and Nuclear Protein Isolation**

After the treatment, kidney tissues or cell samples were subjected to the extraction of cytoplasmic and nuclear proteins. The tissue was pelleted by brief centrifugation, resuspended in ice-cold lysis buffer containing 50 mM Tris-HCl, 100 mM NaCl, 1% Nonidet P-40, 10 mM EDTA, 20 mM NaF, 1 mM PMSF, 3 mM Na3VO4 and protease inhibitor mixture, homogenized thoroughly, and centrifuged (12000 × g for 15 min at 4 °C). The supernatant (cytoplasmic protein) was stored for use after the centrifugation.

In nuclear protein isolation, 250 µl extraction buffer (10 mmol/l Tris-HCL, 10 mmol/l KCl, 5 mmol/l MgCl2, pH 7.6) was added to samples. Then 0.6% Triton X-100 was added to disrupt cell membranes. The tissue samples got sonicated at 4 °C, stayed on ice for 30 min. A total of 250 µl Nuclear Isolation Buffer (10 mmol/l Tris-HCL, 10 mmol/l KCl, 5 mmol/l MgCl2, 0.35 mol/l sucrose) was added then density gradient centrifugation was conducted for 10 min. The supernatant was transferred to another centrifuge tube and four volumes of pre-chilled acetone were added at −20°C and incubated overnight. The supernatant (nuclear protein) was centrifuged at 4°C and 12,000 × g for 30 min. The samples were aliquoted and stored at -80 °C before using for Western blot.

**Western Blotting**

The protein concentrations of each samples were determined using a BCA protein kit (Thermo Fisher Scientific Inc.). Samples with equal amounts of protein (25 µg) were then separated by 10% SDS-PAGE and transferred to polyvinylidene fluoride membranes. Membranes were blocked with 5% non-fat milk for 1 h, then incubated with 1:1,000 dilutions (v/v) of the primary antibodies overnight at 4°C. Primary antibodies were purchased from Cell Signaling Technology, Inc. and included antibodies against Nrf2 (cat. no. 12721; 1:1,000 dilution), p62(cat. no. 5114; 1:1,000 dilution), p-p62 (cat. no. 16177; 1:1,000 dilution), Keap1 (cat. no. 8047; 1:1,000 dilution), Histone H3 (cat. no. 4499; 1:1,000 dilution) and β-actin (cat. no. 4970, 1:2,000 dilution). Subsequently, membranes were exposed to horseradish peroxidase-labeled secondary antibody and incubated for 1 h at 25°C with an enhanced chemiluminescence reagent system (Thermo Fisher Scientific, Inc.). The band of β-actin was used as the loading control. The protein bands were analyzed using ImageJ software (version 1.46; National Institutes of Health, Bethesda, MD, USA).

**Small Interfering (siRNA) Transfection**

The targeted siRNA p62 (sense: 5′-GCAUUGAAGUUGAUAUCGAUU-3′; antisense: 5′-UCGAUAUCAACUUCAAUGCUU-3′) was used in the cell experiment according the published report (Islam et al. 2014). And scrambled siRNA (sense: 5′-CGUACGCGGAAUACUUCGAUU-3′; antisense: 5′-UCGAAGUAUUCCGCGUACGUU-3′) was used as a control unconjugated siRNA in the silencing studies. The cells were transfected with 10 pmol of the indicated siRNAs for 48–72 h using Lipofectamine^TM^ RNAiMax transfection reagent (Invitrogen) according to the manufacturer's protocol. In brief, the cells were seeded into 6-well plate, at 60-80% confluency before transfection. The siRNA (10 pmol) and lipofectamine (9 µL) were diluted in Opti-MeM medium, respectively. Those two media got mixed (1:1) and incubated at 37 ℃ for 5 min, and then added into the cultured cells.

Reference:

Aebi, H. 1984. [13] Catalase in vitro. In *Oxygen Radicals in Biological Systems*, 121-126.

Flohé, L. & Günzler, W. A. 1984. [12] Assays of glutathione peroxidase. In *Oxygen Radicals in Biological Systems*, 114-120.

Islam, M. A., Shin, J.-Y., Yun, C.-H., Cho, C.-S., Seo, H. W., Chae, C., et al. (2014) The effect of RNAi silencing of p62 using an osmotic polysorbitol transporter on autophagy and tumorigenesis in lungs of K-rasLA1 mice. *Biomaterials,* 35(5)**,** 1584-1596.

McCord, J. M. & Fridovich, I. (1969) Superoxide dismutase. An enzymic function for erythrocuprein (hemocuprein). *J Biol Chem,* 244(22)**,** 6049-55.

Ohkawa, H., Ohishi, N. & Yagi, K. (1979) Assay for lipid peroxides in animal tissues by thiobarbituric acid reaction. *Analytical Biochemistry,* 95(2)**,** 351-358.
